# Supplementary material for: Pathogenic and transcriptomic differences of emerging SARS-CoV-2 variants in the Syrian golden hamster model
Source: bioRxiv. 2021 Jul 12:2021.07.11.451964. Preprint. [Version 1] doi: 10.1101/2021.07.11.451964 (PMC8282094; doi:10.1101/2021.07.11.451964)
Supplement: 1 [file NIHPP2021.07.11.451964V1-supplement-1.pdf]

bioRxiv preprint doi: https://doi.org/10.1101/2021.07.11.451964; this version posted July 12, 2021. The copyright holder for this preprint (which was not certified by peer review) is the author/funder. This article is a US Government work. It is not subject to copyright under 17 USC 105 and is also made available for use under a CC0 license.

634

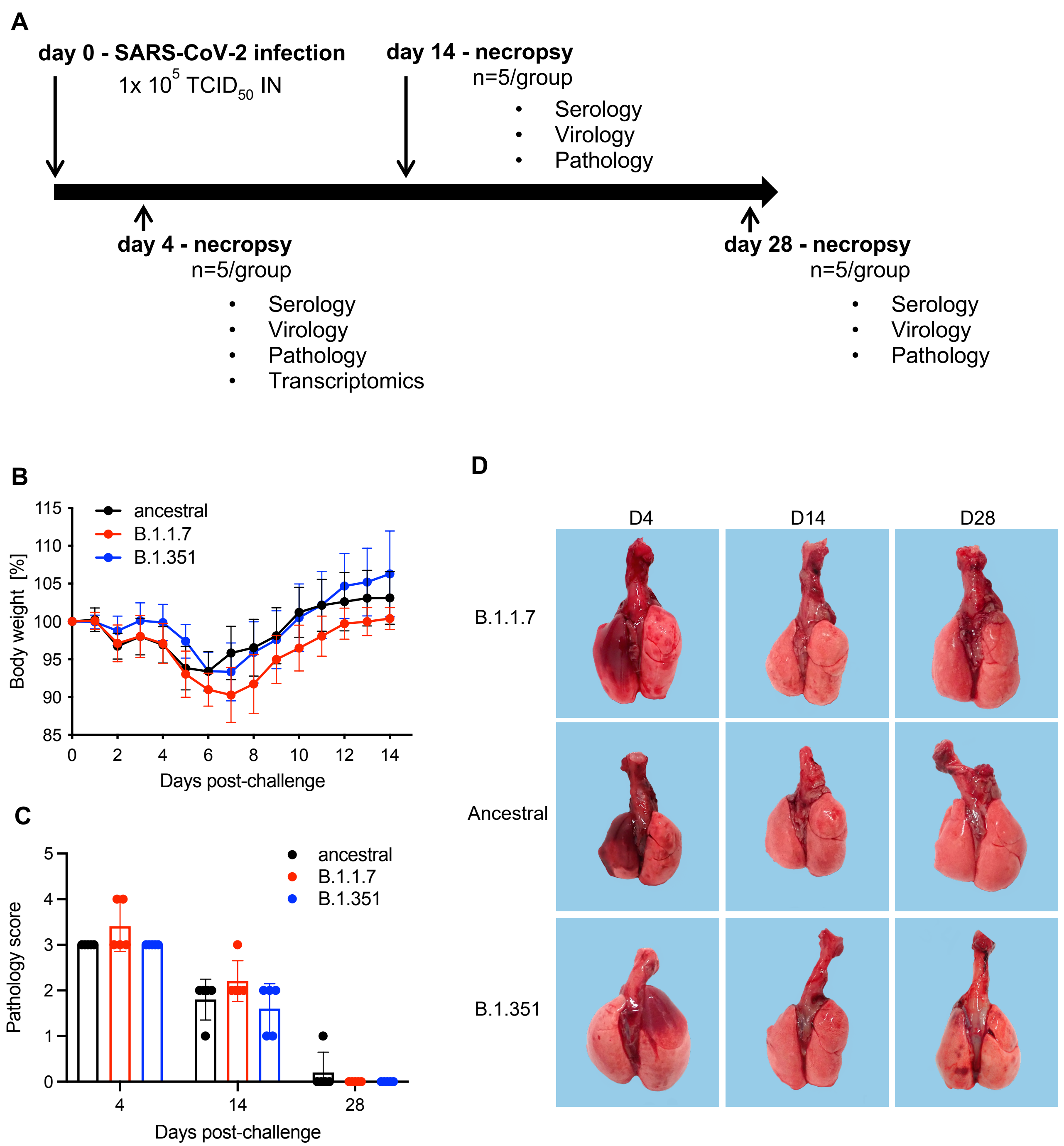

Figure S1. Study outline, body weight changes and pathology of infected hamsters. (A) Schematic study outline. (B) Body weight changes in hamsters (n=10/group). (C) Evidence of interstitial pneumonia was recorded in histopathology samples. (D) Representative pictures of hamster lungs with lesions during disease progression. Gross lung images at day (D) 4 , 14 and 28 post-challenge.

bioRxiv preprint doi: https://doi.org/10.1101/2021.07.11.451964; this version posted July 12, 2021. The copyright holder for this preprint (which was not certified by peer review) is the author/funder. This article is a US Government work. It is not subject to copyright under 17 USC 105 and is also made available for use under a CC0 license.

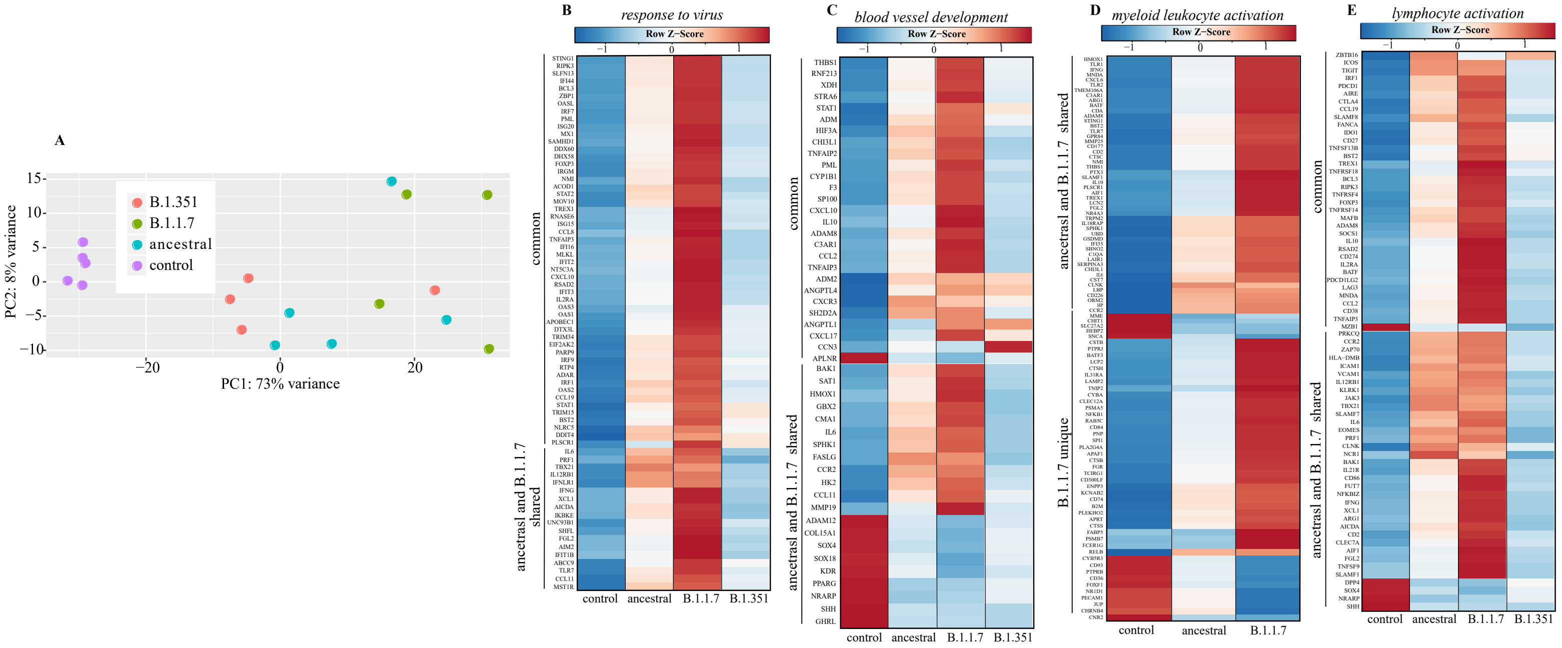

Figure S2. SARS-CoV-2 variants induce distinct transcriptional changes. (A) Principal component analysis of control (n=5) animals and infected animals 4 DPC with ancestral (n=5), B.1.1.7 (n=4) or B.1.351 (n=4) variants. Heatmaps representing DEGs enriching to GO terms from Fig. 5E including (B) “response to virus”, (C) “blood vessel development”, (D) “myeloid leukocyte activation” and (E) lymphocyte activation.” DEGs are either shared among all variant infections or between ancestral and B.1.1.7 variant infections. Columns of all heatmaps represent the average rpkm. Range of colors per each heatmap is based on scaled and centered rpkm values of the represented DEGs. Red represents upregulation; blue, downregulation.

bioRxiv preprint doi: https://doi.org/10.1101/2021.07.11.451964; this version posted July 12, 2021. The copyright holder for this preprint (which was not certified by peer review) is the author/funder. This article is a US Government work. It is not subject to copyright under 17 USC 105 and is also made available for use under a CC0 license.

636

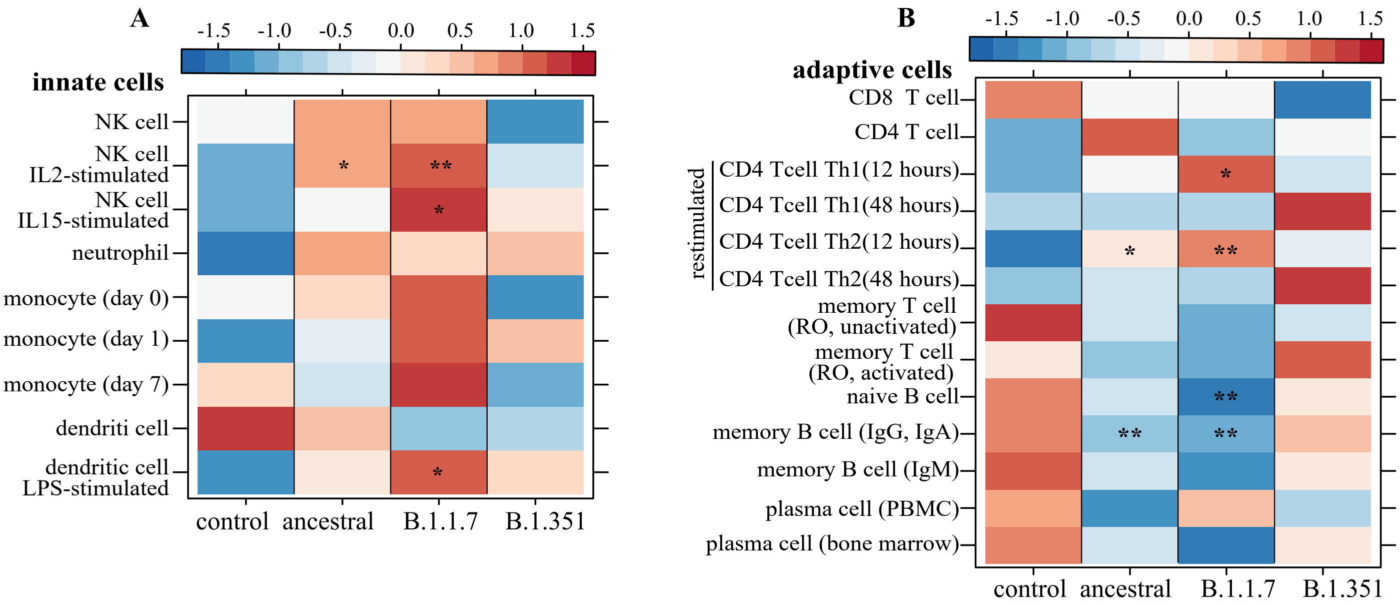

Figure S3. Digital cell quantification in hamster lungs. Heatmaps representing relative changes in (A) innate and (B) adaptive immune cell frequencies using ImmQuant with IRIS database. Each column represents the average relative expression level of the given immune cell. Range of colors per each heatmap is based on scaled and centered relative expression values. Red represents upregulation; blue represents downregulation. Statistical significance is indicated **p < 0.01 and *p < 0.05.
